# Comparison of Chromosome 4 gene expression profile between lung telocytes and other local cell types

**DOI:** 10.1111/jcmm.12746

**Published:** 2015-12-17

**Authors:** Dongli Song, Dragos Cretoiu, Minghuan Zheng, Mengjia Qian, Miaomiao Zhang, Sanda M. Cretoiu, Luonan Chen, Hao Fang, Laurentiu M. Popescu, Xiangdong Wang

**Affiliations:** ^1^ Zhongshan Hospital, Fudan University Center for Clinical BioinformaticsShanghai Institute of Clinical BioinformaticsShanghaiChina; ^2^ Division of Cellular and Molecular Biology and HistologyDepartment of Morphological SciencesCarol Davila University of Medicine and PharmacyBucharestRomania; ^3^ Victor Babeş National Institute of PathologyBucharestRomania; ^4^ State Key Lab of Systems BiologyChinese Academy of ScienceShanghaiChina; ^5^ Department of AnesthesiologyZhongshan Hospital and Jinshan Hospital of Fudan UniversityShanghaiChina

**Keywords:** chromosome 4, telocytes, mesenchymal stem cells, fibroblasts, alveolar type II cells, airway epithelial cells, lymphocytes

## Abstract

Telocytes (TCs) are new cellular entities of mesenchymal origin described almost ubiquitously in human and mammalian organs (www.telocytes.com). Different subtypes of TCs were described, all forming networks in the interstitial space by homo‐ and heterocellular junctions. Previous studies analysed the gene expression profiles of chromosomes 1, 2, 3, 17 and 18 of murine pulmonary TCs. In this study, we analysed by bioinformatics tools the gene expression profiles of chromosome 4 for murine pulmonary TCs and compared it with mesenchymal stem cells (MSCs), fibroblasts (Fbs), alveolar type II cells (ATII), airway basal cells, proximal airway cells, CD8(+) T cells from bronchial lymph nodes (T‐BL) and CD8(+) T cells from lungs (T‐L). Key functional genes were identified with the aid of the reference library of the National Center for Biotechnology Information Gene Expression Omnibus database. Seventeen genes were up‐regulated and 56 genes were down‐regulated in chromosome 4 of TCs compared with other cells. Four genes (Akap2, Gpr153, Sdc3 and Tbc1d2) were up‐regulated between one and fourfold and one gene, Svep1, was overexpressed over fourfold. The main functional networks were identified and analysed, pointing out to a TCs involvement in cellular signalling, regulation of tissue inflammation and cell expansion and movement.

## Introduction

Telocytes (TCs) are newly described cells of the interstitial space 1, 2 which are ubiquitously distributed in mice and humans 3, 4, 5, 6, 7, 8, 9, 10, 11, 12, 13, 14, 15, 16, 17. Telocytes are likely to have a mesenchymal origin 18 and are best characterized by very long extensions called telopodes (Tps) (for details see reviews 17, 19. They were characterized in terms of ultrastructure 20, 21, immunophenotype 22, proteomic 23, gene profile 24, 25, 26 and miRNA imprint 27, 28, 29 and shown to be different from fibroblasts, mesenchymal cells or endothelial cells. Moreover, TCs display distinct electrophysiological properties 30, 31, 32, 33. The very long (tens to hundreds of micrometres) Tps classically described as an alternation of dilated regions—podoms and filamentous regions—podomers, were recently viewed by FIB‐SEM tomography 3D reconstruction 2. Therefore, the real aspect of Tps consists in regions with classical aspect of beads on a string appearance and ‘ribbon‐like’ regions 34.

Telocytes were suggested to participate in intercellular information exchange and interactions by extracellular vesicle release 29, 35. In addition, their secretome might have a modulatory role in stem cell proliferation and differentiation 36. Other hypotheses, plead in favour of a role as progenitor cells during inflammatory/repair processes 37. Telocytes have recently been shown to act as progenitor cells in adulthood, being able to differentiate in cells like interstitial cells of Cajal, myofibroblasts and even in fibroblasts 38. Also, during morphogenesis, it might be possible to behave like inductors/regulators of differentiation for parenchymal cells 38, 39.

Our previous studies identified characters and patterns of TCs‐specific or TCs‐dominated gene profiles in chromosome 1, 2, 3, 17 and 18 using global comparison between TCs and other cell types found in the mouse lung tissue 24, 25, 26. To further study the characters and patterns of TC‐specific or TC‐dominated gene expression profiles, we currently performed a detailed analysis for chromosome 4, and investigated the characteristic gene networks and potential functional association using bioinformatics tools. Pulmonary TCs in cell culture, harvested on day 5 (TC5) and on day 10 (TC10) were compared with mesenchymal stem cells (MSCs), fibroblasts (Fbs), alveolar type II cells (ATII), airway basal cells (ABCs), proximal airway cells (PACs), CD8^+^ T cells from bronchial lymph nodes (T‐BL) and CD8^+^ T cells from lung (T‐L). Key functional genes were identified with the aid of the reference library of the National Center for Biotechnology Information (NCBI) Gene Expression Omnibus database.

## Material and methods

### Isolation and culture

Telocytes were isolated from the lung tissues of mice, primary cultured in a concentration of 1 × 10^5^ cells/cm^2^, and harvested on days 5 (TC5) and on days 10 (TC10), as previously described 28. RNA isolation, preparation, labelling and hybridization for DNA microarray (The Mouse 4 × 44K Gene Expression Array; Agilent, Shanghai, China), we gained about 39,000+ mouse genes and transcripts represented with public domain annotations, according to the protocol of One‐Color Microarray‐Based Gene Expression Analysis. The hybridized arrays were washed, fixed and scanned by the Agilent DNA Microarray Scanner (part number G2505B).

### Data collection and mining

The gene expression profiles of pulmonary TC5 and TC10, Fbs and MSCs were collected from a previous study 28. Gene expression profiles for ATII, ABCs, PACs, T‐BL and T‐L were obtained from the NCBI Gene Expression Omnibus database (GSE6846 40, GSE27379 41, GSE28651 42). The microarray was composed of 45,101 probes. First, we eliminated the probe sets without corresponding official symbol, leaving 39,417 probes and 21,680 genes.

### Identification of differentially expressed genes

The identification of differentially expressed genes was done as the method described in our previous study 24. Briefly, after the acquired data normalized with quantile normalization, the probe level (*_norm_RMA.pair) files and gene level (*_RMA.calls) files were generated. Subsequent data processing was further analysed with Agilent GeneSpring GX software (version 11.5.1) software package and differentially expressed genes were identified through fold change filtering. Hierarchically clustered was performed with the Agilent GeneSpring GX software (version 11.5.1). Gene Ontology analysis and String Network analyses were performed with the standard enrichment computation method to uncover the relevance among variant proteins expressed by variant genes.

Eight‐five per cent of mouse genes (approx. 20,000–25,000 genes) is very similar with the human genes. This study investigates gene expression profiles of chromosome 4 in different lung cell populations to search for TC‐specific regulated genes. Up‐ or down‐regulated folds of TC‐genes were calculated by comparison with other cells and subtracted its own multiple of TC, after the average of gene expression in each cells.

## Results

**Figure 1:**
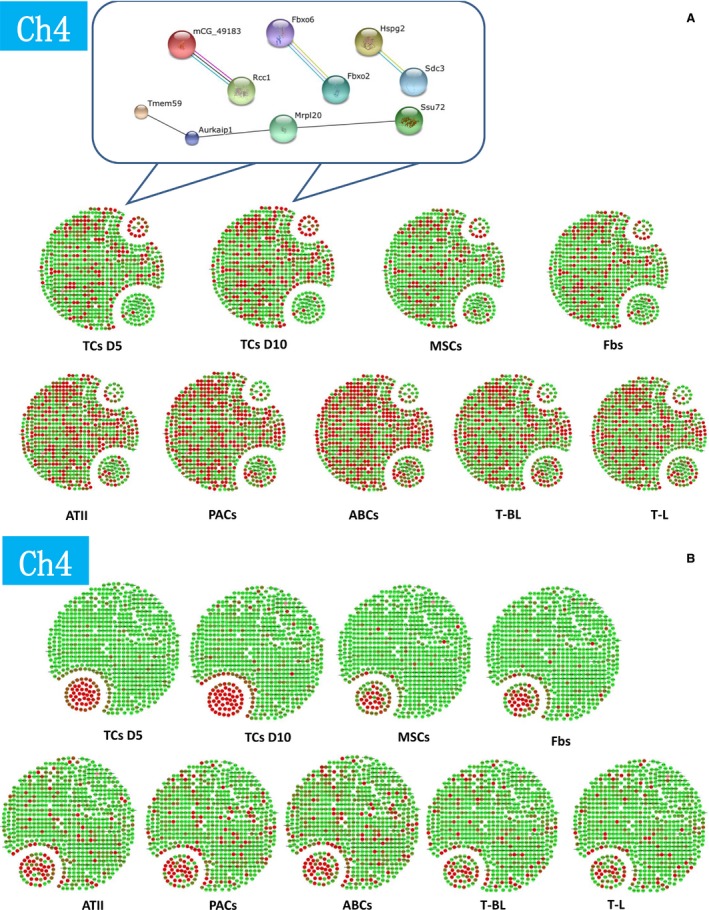
Expression profiles of the selected genes as an active group of chromosome 4 of telocytes (TCs) isolated and cultured from mouse lungs on days 5 (D5) and 10 (D10), as compared with fibroblasts (Fbs), mesenchymal stem cells (MSCs), alveolar type II cells (ATII), airway basal cells (ABCs), proximal airway cells (PACs), CD8^+^ T cells come from bronchial lymph nodes (T‐BL), and CD8^+^ T cells from lung (T‐L) respectively (**A**). The profiles for entire genes are described in Supplementary Document 1. The selected core network and whole mouse network are linked by the documented functional interactions from various databases (see Materials and methods). Genes in each network are indicated in red and some of their nearest neighbours are indicated by dark grey nodes. A group of telocyte genes up‐regulated and down‐regulated more than zerofold as compared with all other cells and existed in telocytes on days 10 and 5 were selected as telocyte‐specific or dominated genes in chromosome 4 (**A**). Top 50 up‐ or down‐regulated genes of each cells were also evaluated and their distribution within chromosome 4 genes showed the difference between cells (**B**). Details of the selected network in each cell type are in Figures S1–S9.

Table 1 presents the global analysis of chromosome 4 genes in lung TCs. We found that 17 genes were up‐regulated and 56 genes were down‐regulated in chromosome 4 of TCs. Among the up‐regulated genes, 12 genes (1700009N14Rik, Aurkaip1, Fam176b, Fbxo6, Hspg2, Macf1, Mast2, Otud3, Plekhm2, Tm2d1, Tmem59, Zcchc17) were overexpressed between zero and onefold (Table 1A), 4 genes (Akap2, Gpr153, Sdc3, Tbc1d2) were up‐regulated between one and fourfold (Table 1B) and one gene, Svep1, was overexpressed over fourfold, in both TC D5 and TC D10, as compared with other cells (Table 1C). The genes highly expressed in TC5 were similar with those in TC10 and different from MSCs, Fbs, ATII, ABCs, PACs, T‐BL or T‐L. The direct (physical) and indirect (functional) relationships, including associations, of these genes were analysed by String Network analysis and the interactions and potential functional links between these genes are displayed in Figure 1.

**Table 1:** Summary of up‐regulated genes in TCs, as compared with others. (A) Genes up‐regulated between zero and onefold in TCs as compared with others. (B) Genes up‐regulated between one and fourfold in TCs as compared with others. (C) Genes up‐regulated >fourfold in TCs as compared with others

Compared pairs/fold up‐regulated	>0	>1	>4
TC5 *versus* others	51	13	3
TC10 *versus* others	34	8	1
TCs *versus* others	17	5	1

Gene symbol	Folds (TC5 *versus* others/TC10 *versus* others)						
	Fibroblast	Stem	ATII	CD8_T_BL	CD8_T_LL	Basal_cell	Duct_cell
(A)							
1700009N14Rik	−0.98/−0.99	−0.97/−0.97	−0.72/−0.8	−0.41/−0.6	−0.23/−0.47	−0.47/−0.64	−0.49/−0.65
Aurkaip1	−0.37/−0.09	−0.46/−0.22	−0.35/−0.32	−0.43/−0.42	−0.48/−0.47	−0.61/−0.6	−0.51/−0.5
Fam176b	−0.73/−0.73	−0.86/−0.86	−0.09/−0.34	−0.36/−0.55	−0.56/−0.69	−0.88/−0.92	−0.94/−0.96
Fbxo6	−0.33/−0.17	−0.56/−0.45	−0.6/−0.64	−0.83/−0.85	−0.89/−0.9	−0.77/−0.8	−0.84/−0.86
Hspg2	−0.62/−0.7	−0.21/−0.38	−0.69/−0.82	−0.7/−0.83	−0.6/−0.77	−0.76/−0.87	−0.84/−0.91
Macf1	−0.74/−0.66	−0.5/−0.35	−0.64/−0.67	−0.52/−0.56	−0.47/−0.51	−0.65/−0.68	−0.46/−0.51
Mast2	−0.48/−0.15	−0.62/−0.38	−0.92/−0.9	−0.96/−0.95	−0.95/−0.94	−0.81/−0.79	−0.87/−0.85
Otud3	−0.61/−0.5	−0.79/−0.73	−0.4/−0.43	−0.09/−0.16	−0.18/−0.24	−0.12/−0.2	−0.11/−0.19
Plekhm2	−0.36/−0.6	−0.17/−0.49	−0.46/−0.76	−0.51/−0.78	−0.47/−0.77	−0.77/−0.9	−0.72/−0.87
Tm2d1	−0.32/−0.14	−0.43/−0.27	−0.39/−0.44	−0.27/−0.35	−0.36/−0.42	−0.28/−0.35	−0.23/−0.31
Tmem59	−0.51/−0.43	−0.45/−0.36	−0.99/−0.99	−0.99/−0.99	−0.99/−0.99	−1/−1	−1/−1
Zcchc17	−0.53/−0.45	−0.67/−0.61	−0.54/−0.61	−0.08/−0.23	−0.59/−0.65	−0.62/−0.69	−0.76/−0.8
(B)							
Akap2	−0.89/−0.81	−0.73/−0.54	−0.78/−0.73	−0.78/−0.74	−0.82/−0.78	−0.79/−0.75	−0.82/−0.78
Gpr153	−0.93/−0.92	−0.66/−0.61	−0.67/−0.72	−0.98/−0.99	−0.96/−0.97	−0.98/−0.99	−0.92/−0.93
Sdc3	−0.74/−0.62	−0.88/−0.83	−0.65/−0.62	−0.84/−0.83	−0.73/−0.71	−0.79/−0.78	−0.87/−0.87
Tbc1d2	−0.91/−0.78	−0.99/−0.97	−0.78/−0.6	−0.99/−0.98	−0.97/−0.94	−0.8/−0.65	−0.94/−0.9
(C)							
Svep1	−0.97/−0.97	−0.84/−0.83	−0.9/−0.92	−0.95/−0.96	−0.95/−0.97	−0.95/−0.96	−0.94/−0.95

Among the down‐regulated genes, 54 genes were expressed zero and onefold in TCs than in other cells (Table 2A) and 2 genes, Masp2 and Rngtt (Table 2B) were one to fourfold lower in TCs than in other cells.

**Table 2:** Summary of down‐regulated genes in TCs, as compared with others. (A) Genes down‐regulated between zero and onefold in TCs as compared with others. (B) Genes down‐regulated between one and fourfold in TCs as compared with others

Compared pairs/fold down‐regulated	>0	>1	>4
TC5 *versus* others	70	3	0
TC10 *versus* others	142	10	0
TCs *versus* others	56	2	0

Gene symbol	Folds (TC5 *versus* others/TC10 *versus* others)						
	Fibroblast	Stem	ATII	CD8_T_BL	CD8_T_LL	Basal_cell	Duct_cell
(A)							
1700013G24Rik	0.18/0.28	0.34/0.45	1.43/0.93	0.39/0.07	5.42/4.01	50.81/38.78	22.78/17.36
2210012G02Rik	1.36/0.8	0.68/0.28	31.01/16.83	100.21/53.77	56/30.27	44.47/23.55	21.26/11.08
2610301B20Rik	1.63/1.75	1.3/1.41	7.41/5.43	4.38/2.99	0.97/0.48	3.12/2.05	1.17/0.61
2610528B01Rik	0.66/0.34	15.64/12.46	17.59/9.98	49.88/28.21	12.65/6.94	31.04/17.35	15.34/8.41
4930535I16Rik	10.92/6.52	0.98/0.25	11.07/4.56	1.28/0.02	2/0.36	17.05/7.07	18.12/7.59
5430416O09Rik	0.44/0.78	0.21/0.5	22.22/19.97	1.13/0.87	1.01/0.79	3.05/2.55	1.3/1.03
9430015G10Rik	0.98/1.38	0.12/0.35	7.93/6.85	34.35/29.18	26.31/22.64	20.37/17.21	12.22/10.32
9930104L06Rik	0.4/0.68	0.4/0.68	0.43/0.25	2.83/2.26	2.36/1.9	8.44/7.02	1.86/1.44
AA415398	0.6/0.16	0.38/0.01	8.93/4.29	3.07/1.11	8.47/3.97	20.5/10.11	17.32/8.52
Agmat	0.35/0.57	0.09/0.27	3.74/3.04	4.14/3.26	7.97/6.53	19.18/15.67	13.9/11.38
Anp32b	0.43/0.8	1.35/1.96	1.59/1.39	2.42/2.07	1.88/1.62	14.09/12.48	14.37/12.8
BC057079	0.14/0.46	0.33/0.7	3.03/2.77	7.53/6.75	6.72/6.1	3.87/3.41	6.1/5.47
Btf3l4	0.49/1.07	0.11/0.53	2.78/2.84	2.63/2.58	1.29/1.29	4.31/4.22	3.69/3.63
C430048L16Rik	1.28/2.16	0.75/1.43	0.23/0.25	1.2/1.17	2.54/2.53	3.67/3.59	1.08/1.06
Cap1	3.57/6.8	3.03/5.89	0.2/0.5	1.47/1.99	0.48/0.81	2.88/3.7	3.74/4.77
Casp8ap2	0.1/0.45	0.29/0.7	3.9/3.71	38.04/35.47	51.64/48.86	4.79/4.4	1.56/1.4
Ccnl2	0.52/1.57	0.07/0.8	1.09/1.57	16.55/20.03	15.86/19.48	4.59/5.69	3.58/4.51
Chd5	0.87/0.28	7.33/4.71	21.45/10.24	40.34/19.1	36.74/17.61	69.19/33.06	27.74/13.02
Clcnkb	1.03/1.24	1.08/1.3	12.27/9.73	1.13/0.67	0.44/0.15	8.62/6.54	4.45/3.29
Col16a1	0.43/0.3	3.97/3.52	2.13/1.08	1.25/0.45	2.43/1.25	4.61/2.62	2.81/1.47
Cyp4a31	7.9/17.22	5.85/13.02	0.04/0.56	0.8/1.62	1.47/2.64	7.54/11.38	0.96/1.86
Dennd4c	0.44/0.82	0.06/0.34	167.81/154.43	223.55/199.84	388.03/351.76	177.75/158.54	132.18/118.48
Dnajc11	0.04/0.35	0.16/0.51	7.39/6.99	11.28/10.37	4.03/3.71	5.72/5.21	4.66/4.25
Eif2b3	0.31/0.56	0.72/1.05	6.17/5.24	4.11/3.32	2.29/1.82	7.03/5.77	7.08/5.85
Gja10	0.28/1.02	0.2/0.9	0.91/1.21	1.69/2.01	7.08/8.17	7.59/8.6	2.81/3.28
Gng10	0.15/0.05	0.94/0.77	1.94/0.97	17.25/10.86	10.63/6.66	3.4/1.85	2.47/1.26
Gpr3	0.71/1.32	0.61/1.18	0.55/0.54	0.9/0.83	2.68/2.59	6.6/6.3	2.08/1.97
Guca2b	0.49/1.03	0.39/0.89	6.42/6.38	20.5/19.76	0.41/0.38	1.58/1.49	1.93/1.83
Htr6	0.05/0.32	0.11/0.39	2.89/2.55	6.02/5.23	3.42/2.98	17.29/15.2	7.1/6.21
Itgb3bp	1.69/3.05	0.19/0.8	0.32/0.46	1.37/1.54	1.09/1.27	2.05/2.26	2.07/2.29
Lrp8	1.39/1.05	0.33/0.14	1.94/0.85	3.72/1.88	8.57/4.92	8.29/4.66	6.82/3.79
Mdn1	0.05/0.2	4.47/5.26	2.63/2.04	22.03/17.71	17.2/13.99	3.22/2.42	3.95/3.03
Mrpl50	1.02/1.86	0.62/1.3	1.23/1.32	1.47/1.49	0.87/0.91	5.43/5.46	3.48/3.53
Mysm1	0.25/0.63	0.1/0.43	1.13/1.03	5.56/5.09	9.49/8.87	1.59/1.4	0.57/0.46
Nfx1	0.43/0.7	0.47/0.75	6.99/5.95	12.45/10.37	7.26/6.08	16.28/13.58	11.53/9.63
Padi1	2.1/2.28	0.69/0.79	1.6/1.01	3.92/2.7	1.26/0.72	53.37/39.8	39.89/29.84
Pnrc2	0.51/0.96	0.52/0.96	0.52/0.44	2.07/1.82	1.01/0.87	4.13/3.69	2.29/2.02
Ppie	0.2/0.42	0.02/0.21	8.3/7.04	25.22/21.02	12.55/10.53	12.31/10.15	9.68/7.99
Ppp1r8	0.03/0.39	0.59/1.14	14.59/14.36	16.61/15.85	8.84/8.55	9.57/9.09	12.5/11.96
Prpf4	0.29/0.99	1.69/3.16	2.13/2.53	2.49/2.83	2.09/2.44	2.16/2.46	2.69/3.06
Psip1	0.7/1.66	1.75/3.31	0.03/0.18	6.21/7.02	4.28/4.95	0.41/0.56	0.73/0.93
Rbm12b	0.85/1.48	0.01/0.36	0.77/0.74	3.16/2.96	1.96/1.86	42.69/40.57	21.65/20.66
Rere	0.04/0.53	0.03/0.53	114.9/123.97	372.04/389.75	288.69/306.63	246.05/257.22	213.01/223.85
Sit1	0.93/1.91	0.02/0.55	18.38/20.41	58.46/62.81	54.15/59.01	8.62/9.3	3.99/4.38
Slc1a7	0.2/0.6	1.06/1.75	5.81/5.62	1.34/1.21	6.5/6.18	6.15/5.74	2.64/2.45
Slc24a2	0.97/0.64	0.33/0.11	9.22/5.23	3.07/1.41	8.6/4.76	12.23/6.81	18.21/10.41
Smpdl3b	0.11/0.34	1.56/2.08	8.18/7.07	2.69/2.16	7.17/6.08	2.72/2.17	3.6/2.94
Snip1	0.2/0.24	0.14/0.17	23.93/17.75	54.13/39.28	68.21/50.28	13.91/9.87	7.79/5.45
Tle1	0.01/0.37	0.21/0.65	4.58/4.54	1.34/1.26	1.77/1.71	0.43/0.37	0.85/0.79
Trim14	0.45/0.52	0.26/0.32	19.25/14.53	112.18/83.33	51.76/38.86	21.63/15.83	3.67/2.49
Txndc12	2.21/2.33	0.22/0.26	10.57/7.78	4.87/3.33	1.62/0.96	7.28/5.08	6.21/4.33
Ubxn11	4.39/4.71	0.2/0.27	12.07/9.12	21.95/16.25	12.9/9.6	9.9/7.17	4.79/3.37
Usp1	0.72/1.35	0.1/0.51	0.77/0.78	5.14/4.97	3.1/3.04	1.59/1.51	1.81/1.74
Wwp1	0.01/0.12	0.27/0.41	43.21/34.92	46.36/36.37	61.11/48.69	81.23/63.75	83.25/65.68
(B)							
Masp2	13.13/11.07	3.8/3.1	9.4/5.49	23.91/14.1	38.75/23.43	27.31/16.13	20.68/12.18
Rngtt	1.03/1.48	1.25/1.75	1.26/1.02	7.58/6.44	4.49/3.83	2.87/2.35	2.3/1.87

**Figure 2:**
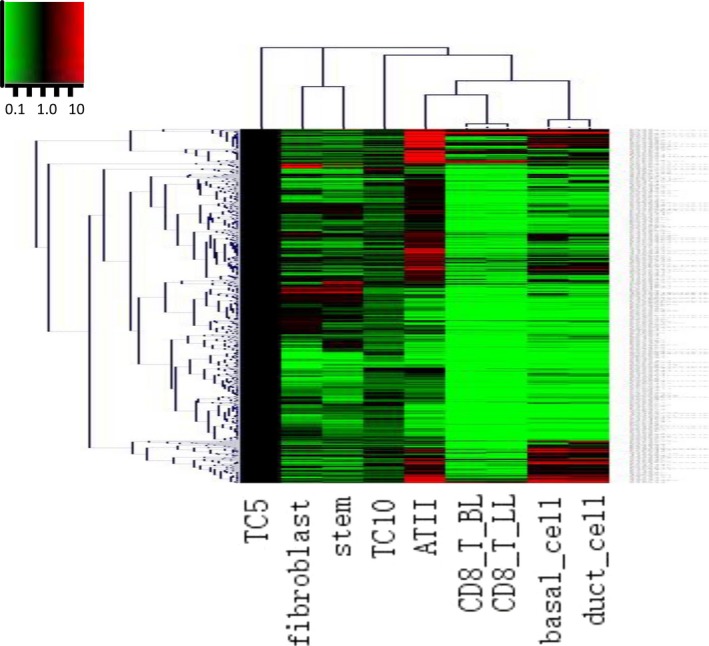
Hierarchical cluster analysis of the differentially expressed genes on chromosomes 4 among telocytes (TCs), mesenchymal stem cells (MSCs), fibroblasts (Fbs), lymphocytes from lungs (T‐LL) and from bronchial lymph nodes (T‐BL), alveolar type II cells (ATII), proximal airway cells (PAC) and airway basal cells (ABC). The differences are described by fold changes and the expression value of genes in TC5 are controls.

Details of up‐ or down gene variations of chromosome 4 were listed in Table S1. The hierarchical cluster plot of the differentially expressed genes illustrated as coded colours (Fig. 2) clearly shows that TCs are less related with the other cells.

Table 3 presents a set of genes were found specifically up‐ or down‐regulated in pulmonary TCs, as compared with Fbs, MSCs, ATII, ABCs, PACs, T‐BL or T‐L respectively. A set of genes up‐ or down‐regulated more than onefold in TC5 were 233 or 49, 249 or 46, 78 or 408, 123 or 378, 125 or 375, whereas the genes up‐ or down‐regulated more than onefold in TC10 were 163 or 92, 164 or 94, 71 or 410, 133 or 372 and 123 or 368.

**Table 3:** The number of genes specifically up‐ or down‐regulated in pulmonary TCs as compared with other cells respectively

Compared pairs	Up >0	Up >1	Up >4	Down >0	Down >1	Down >4
TC10 *versus* fibroblast	367	163	53	353	92	23
TC5 *versus* fibroblast	510	233	69	210	49	12
TCs *versus* fibroblast	354	149	45	197	42	12
TC10 *versus* stem	425	164	43	295	94	19
TC5 *versus* stem	551	249	59	169	46	11
TCs *versus* stem	419	144	33	163	45	11
TC10 *versus* ATII	171	71	17	549	410	229
TC5 *versus* ATII	174	78	20	546	408	225
TCs *versus* ATII	147	61	12	522	383	201
TC10 *versus* CD8BL	225	133	60	495	372	201
TC5 *versus* CD8BL	229	123	65	491	378	205
TCs *versus* CD8BL	204	110	52	470	346	181
TC10 *versus* CD8LL	208	123	56	512	368	194
TC5 *versus* CD8LL	217	125	59	503	375	208
TCs *versus* CD8LL	185	107	50	480	342	178
TC10 *versus* basal cell	128	57	16	592	497	308
TC5 *versus* basal cell	131	57	20	589	499	316
TCs *versus* basal cell	111	44	13	572	472	287
TC10 *versus* duct cell	156	85	32	564	464	267
TC5 *versus* duct cell	155	82	33	565	461	271
TCs *versus* duct cell	144	69	27	553	436	239

## Discussion

Mouse genome is extremely valuable for research since the human and mouse genomes are remarkably similar not only in the structure of their chromosomes but also at the level of DNA sequence. Chromosome 4 represents more than 6 per cent of the total DNA in cells and likely contains 1000–1100 genes 43. In humans, many genetic disorders stemming from chromosome 4 genes are described, *e.g*. achondroplasia, facioscapulohumeral muscular dystrophy, Huntington's disease, to name but a few. Mouse chromosome 4 has a total number of genes of 2430 which encode a number of 1270 proteins.

This study was dedicated to the global analysis of chromosome 4 genes of lung TCs compared with Fbs, MSCs, ATII, ABCs, PACs, T‐BL and T‐L of which 720 genes were measured by bioinformatics tools. We found that 17 genes were up‐regulated and 56 genes were down‐regulated in chromosome 4 of TCs as compared with other cell types.

Four genes, Akap2, Gpr153, Sdc3, Tbc1d2, were found to be more than onefold up‐regulated in TCs as compared with other cell types. Akap2 (A‐kinase (PRKA) anchor protein 2) gene encodes a protein involved in signalling pathways (G Protein signalling pathways and signal transduction PKA) and in modulation of actin filament dynamics 44, 45. Gpr153 (G protein‐coupled receptor 153) gene encodes an orphan receptor with elusive functions 46. Sdc3 (syndecan 3) gene encodes a cell surface proteoglycan (heparan sulphate) involved in the organization of cell shape by affecting the actin cytoskeleton, possibly by transferring signals from the cell surface which seems to have a selectively pro‐inflammatory function 47. Tbc1d2 (TBC1 domain family member 2A) gene encodes a protein found in cell junctions and cytoplasmic vesicles and is apparently involved in positive regulation of GTPase activity and vesicle trafficking 48. Svep1 (sushi, von Willebrand factor type A, EGF and pentraxin domain containing 1) gene encodes a protein involved in cell adhesion 49. Small GTP ases regulate intracellular trafficking (budding, transport and fusion of vesicles) 50 and also intervene in cytoskeletal remodelling, migration and adhesion events 51. Therefore, all these up‐regulated genes encode proteins involved in cell signalling pathways and cytoskeleton organization and imply that TCs could integrate signals and auto‐regulate its own fate, integrating autophagy with endocytic trafficking 52. Moreover, since there are no data regarding the involvement of these four genes in any pulmonary pathology, the precise significance of those up‐regulated genes in TCs still remains unclear.

Among the down‐expressed genes in TCs, Masp2 (mannan‐binding lectin serine peptidase 2) and Rngtt (RNA guanylyltransferase and 5′‐phosphatase) genes were one to fourfold lower comparative with other cells.

## Conclusion

Our data showed, by global analyses, that 73 TCs‐specific or dominant genes in chromosome 4 are different from other lung tissue resident cells or immune migrated cells. Current findings are supportive for our previous studies of TC‐specific gene profiles and potential functional correlations, pointing out the same suggested roles for TCs 24, 25, 26. Thus, TCs appear once more to have a significant role in cellular signalling, regulation of tissue inflammation, and cell expansion and movement.

## Conflicts of interest

The authors declare that they have no competing interests.

## Supporting information


**Figure S1** Details of the selected core network genes in telocytes isolated from the mouse lung and cultured for 10 days in chromosome 4.

**Figure S2** Details of the selected core network genes in telocytes isolated from the mouse lung and cultured for 5 days in chromosome 4.

**Figure S3** Details of the selected core network genes in mouse mesenchymal stem cells in chromosome 4.

**Figure S4** Details of the selected core network genes in mouse fibroblasts in chromosome 4.

**Figure S5** Details of the selected core network genes in mouse alveolar type II cells in chromosome 4.

**Figure S6** Details of the selected core network genes in mouse proximal airway cells in chromosome 4.

**Figure S7** Details of the selected core network genes in mouse airway basal cells in chromosome 4.

**Figure S8** Details of the selected core network genes in mouse CD8^+^ T cells come from bronchial lymph nodes in chromosome 4.

**Figure S9** Details of the selected core network genes in mouse CD8^+^ T cells from lung in chromosome 4.
