# Supplementary figures and images for: Comparison of Chromosome 4 gene expression profile between lung telocytes and other local cell types
Source: J Cell Mol Med. 2015 Dec 17;20(1):71–80. doi: 10.1111/jcmm.12746 (PMC4717865; doi:10.1111/jcmm.12746)

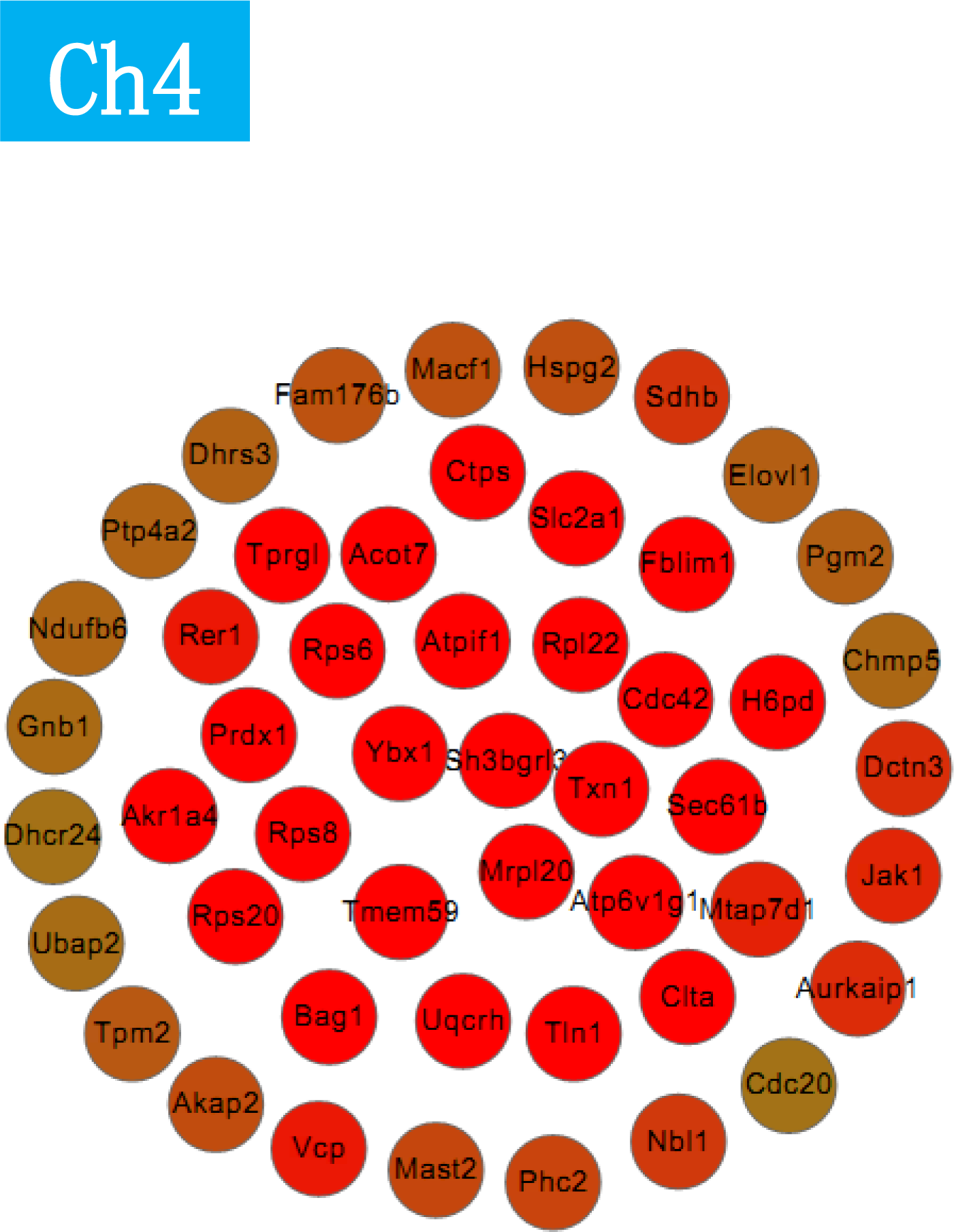

Supplement: Supplementary file 1 — Figure S1 Details of the selected core network genes in telocytes isolated from the mouse lung and cultured for 10 days in chromosome 4. [file JCMM-20-071-s001.tif]

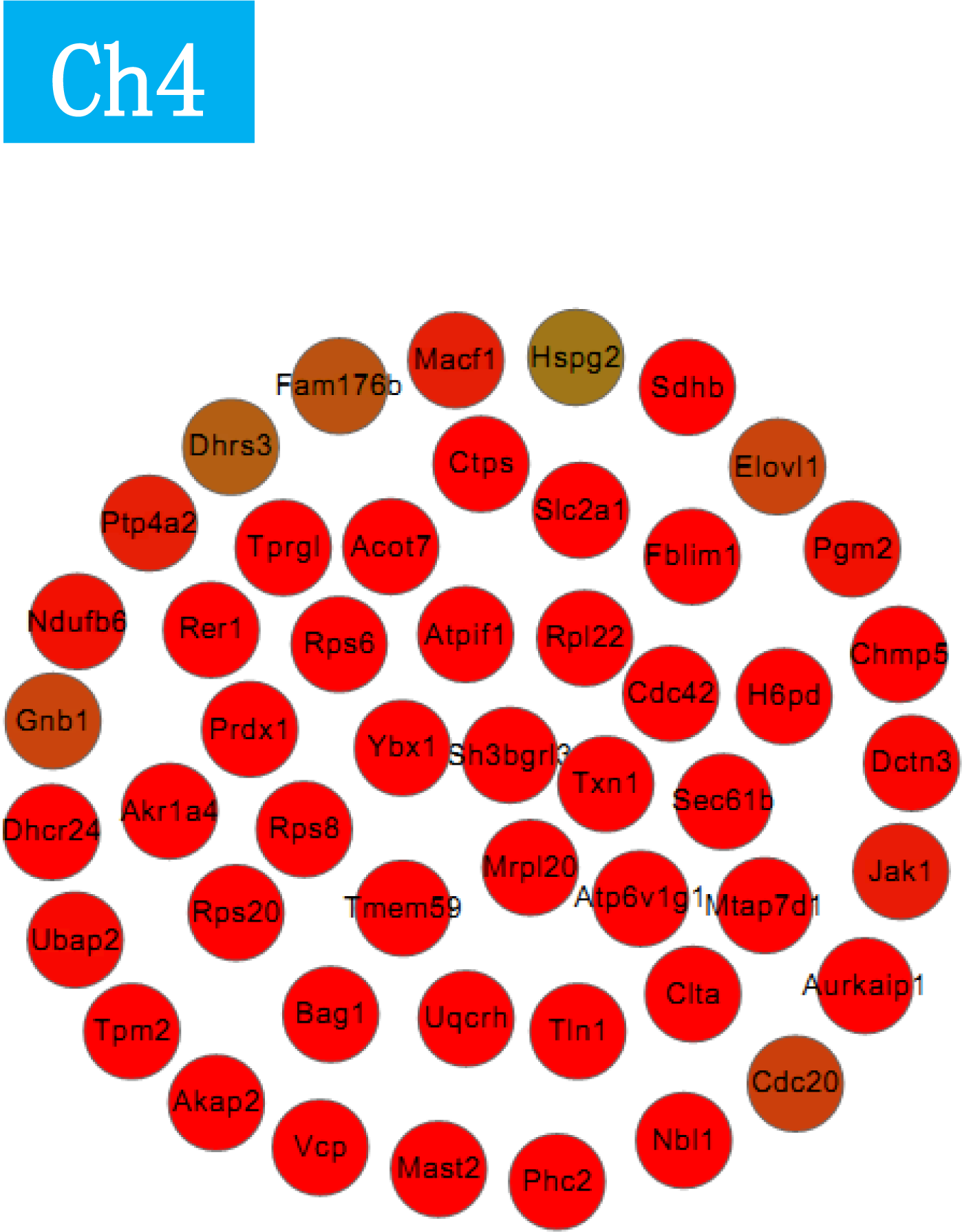

Supplement: Supplementary file 2 — Figure S2 Details of the selected core network genes in telocytes isolated from the mouse lung and cultured for 5 days in chromosome 4. [file JCMM-20-071-s002.tif]

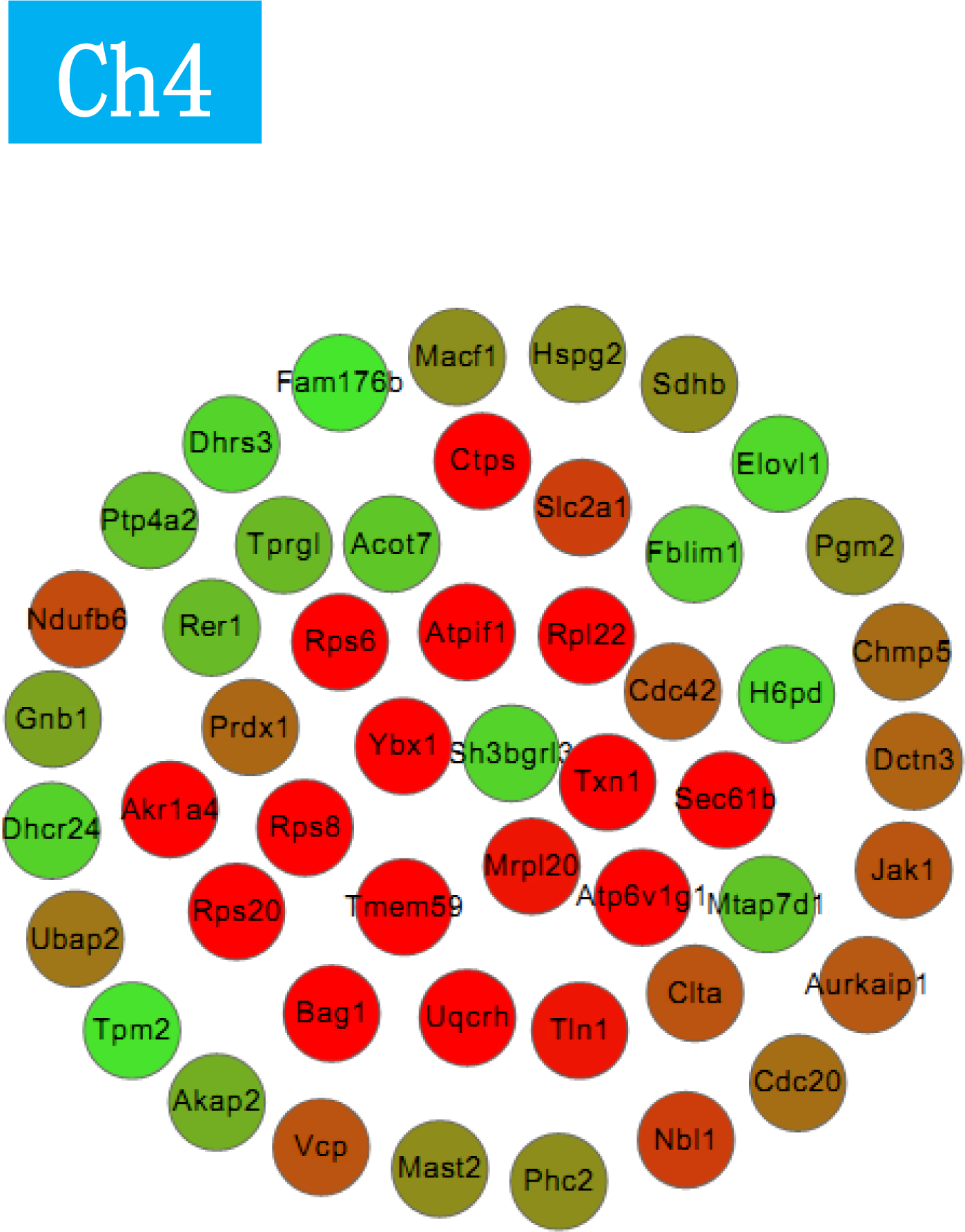

Supplement: Supplementary file 3 — Figure S3 Details of the selected core network genes in mouse mesenchymal stem cells in chromosome 4. [file JCMM-20-071-s003.tif]

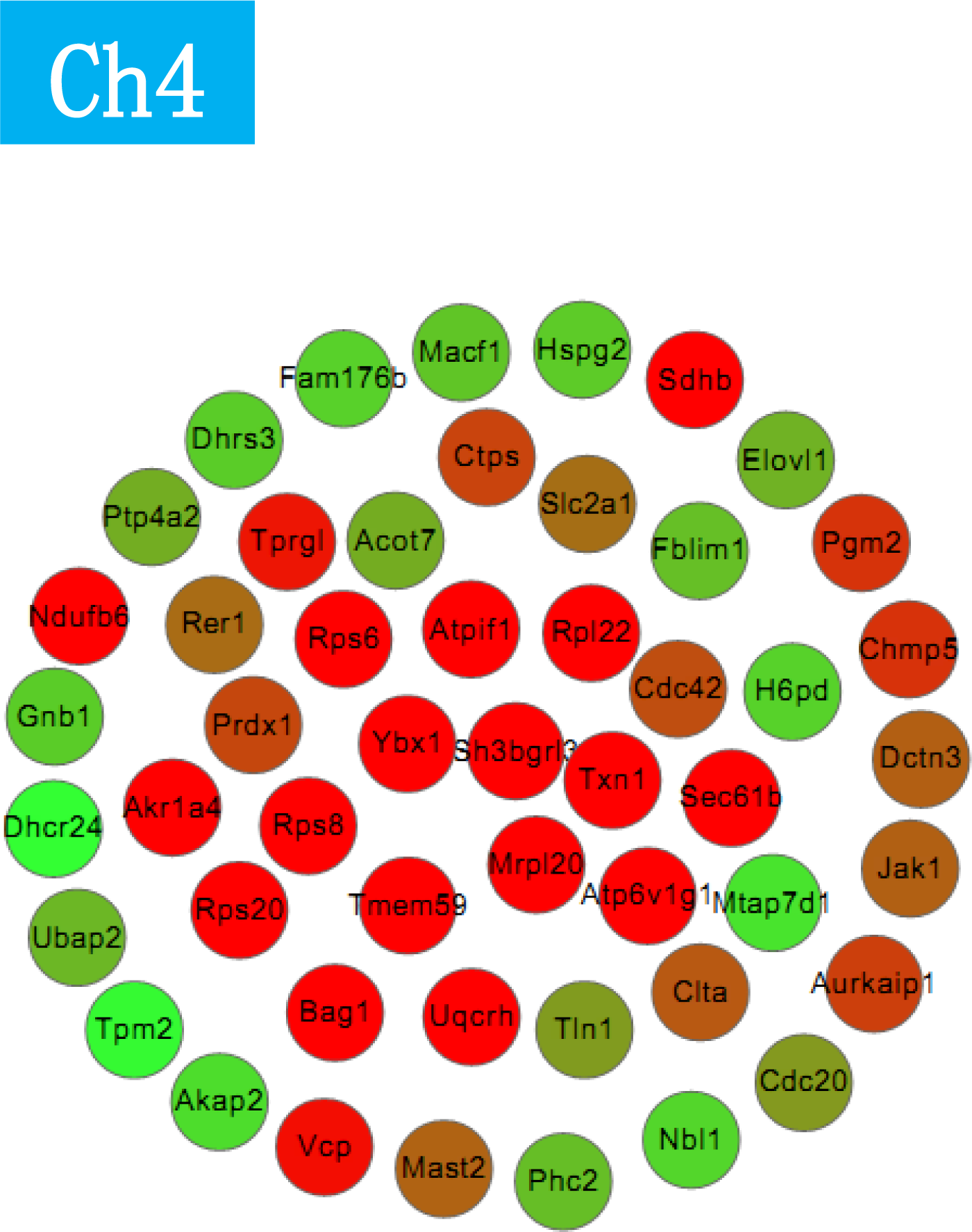

Supplement: Supplementary file 4 — Figure S4 Details of the selected core network genes in mouse fibroblasts in chromosome 4. [file JCMM-20-071-s004.tif]

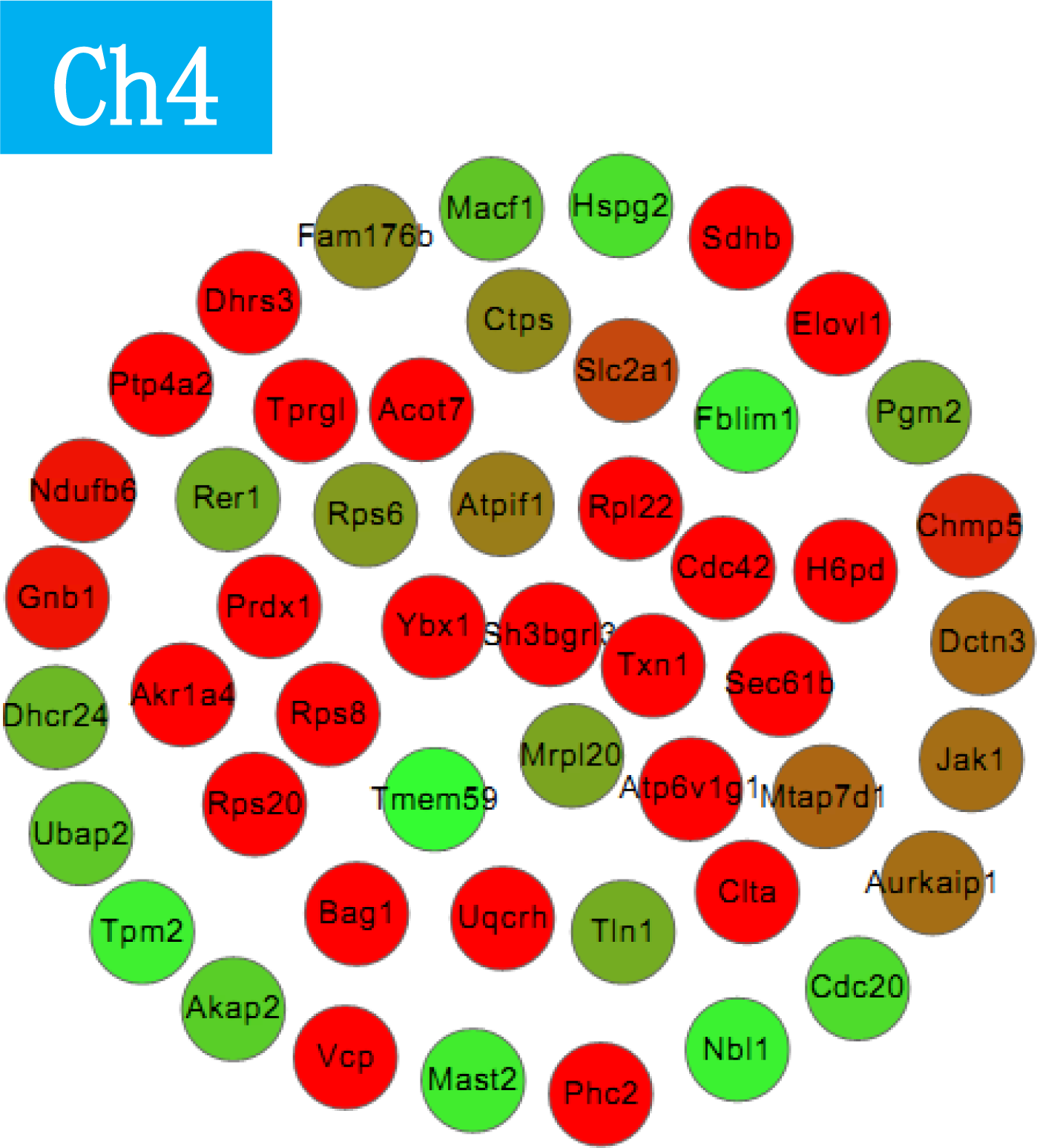

Supplement: Supplementary file 5 — Figure S5 Details of the selected core network genes in mouse alveolar type II cells in chromosome 4. [file JCMM-20-071-s005.tif]

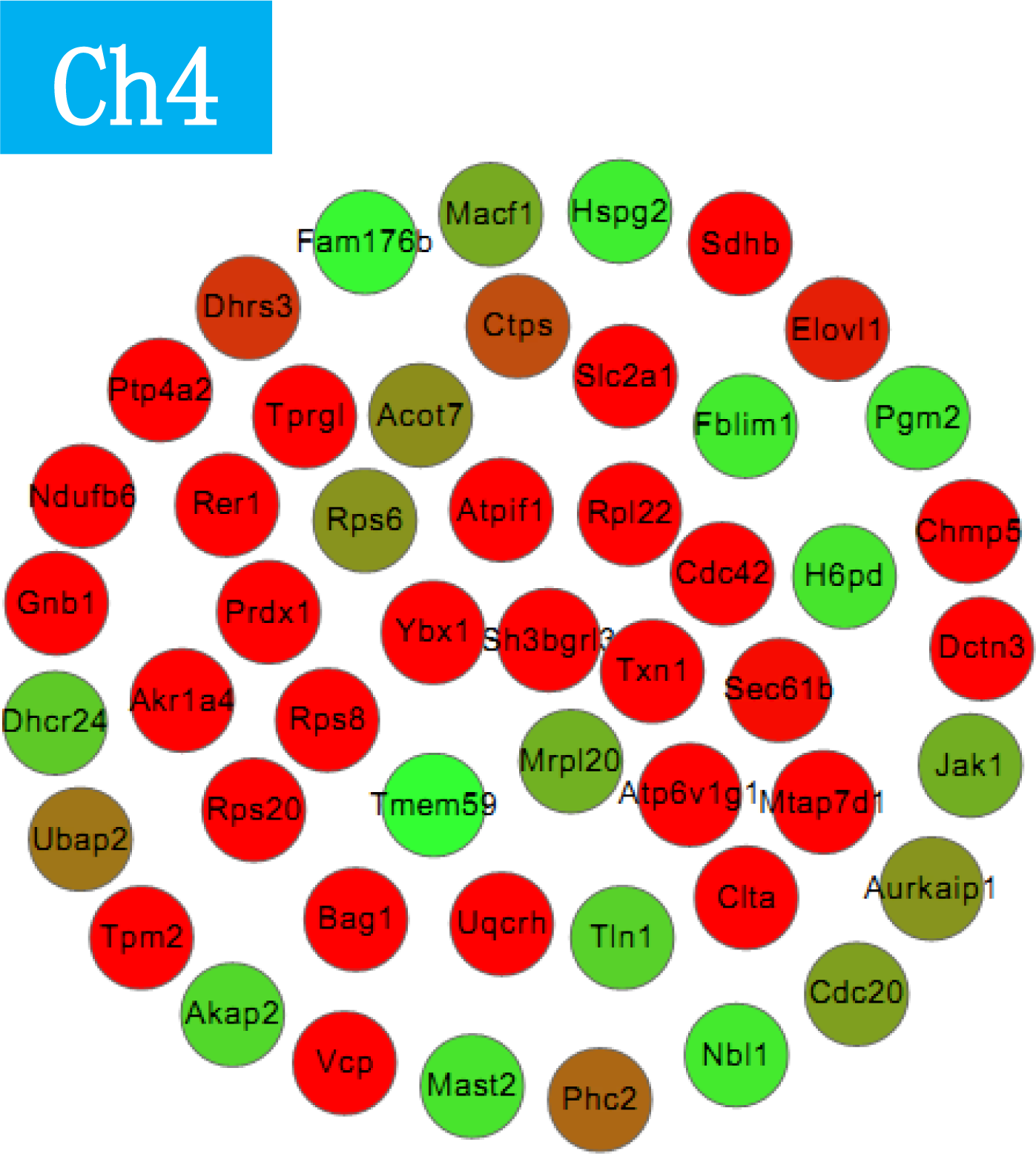

Supplement: Supplementary file 6 — Figure S6 Details of the selected core network genes in mouse proximal airway cells in chromosome 4. [file JCMM-20-071-s006.tif]

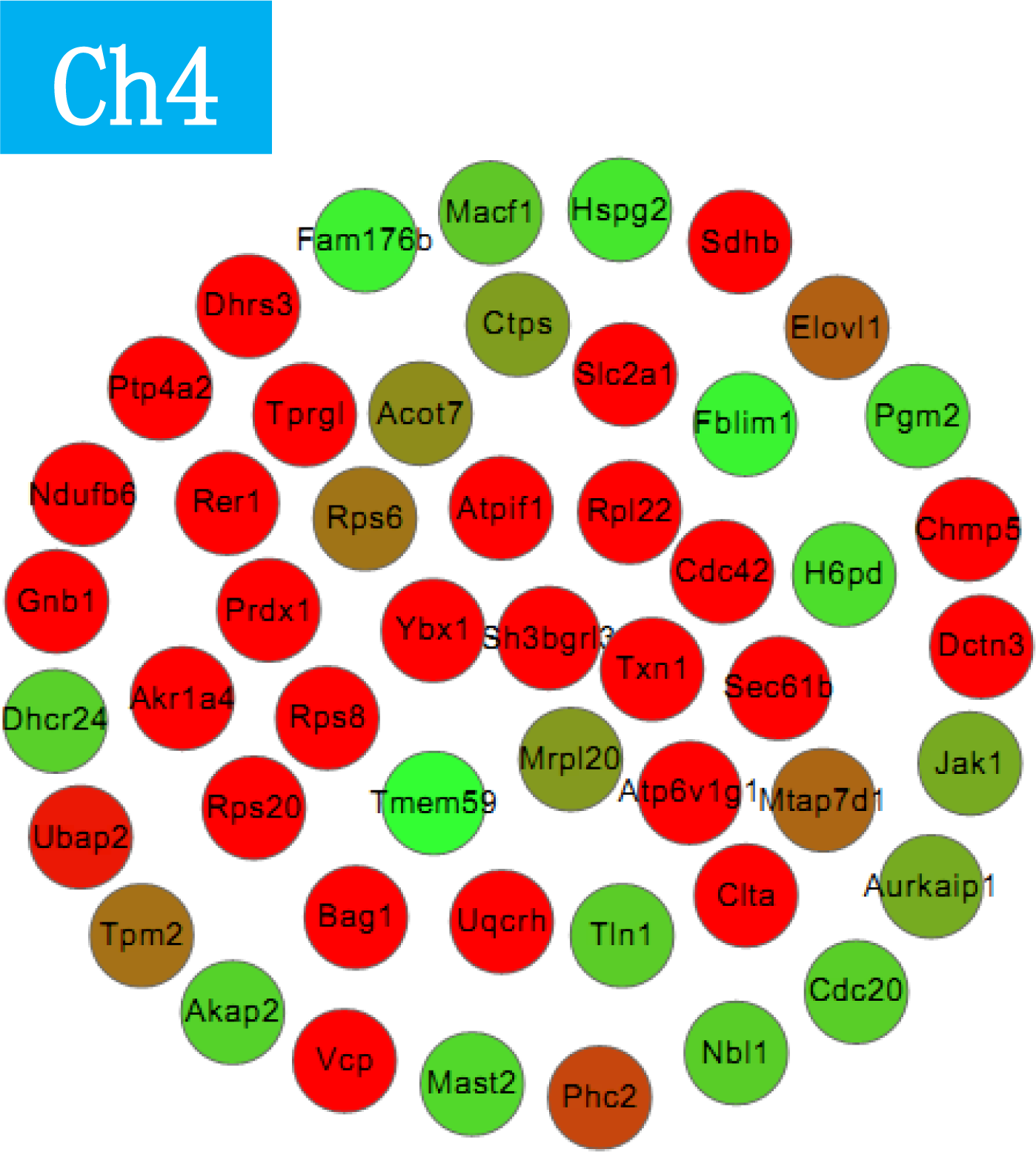

Supplement: Supplementary file 7 — Figure S7 Details of the selected core network genes in mouse airway basal cells in chromosome 4. [file JCMM-20-071-s007.tif]

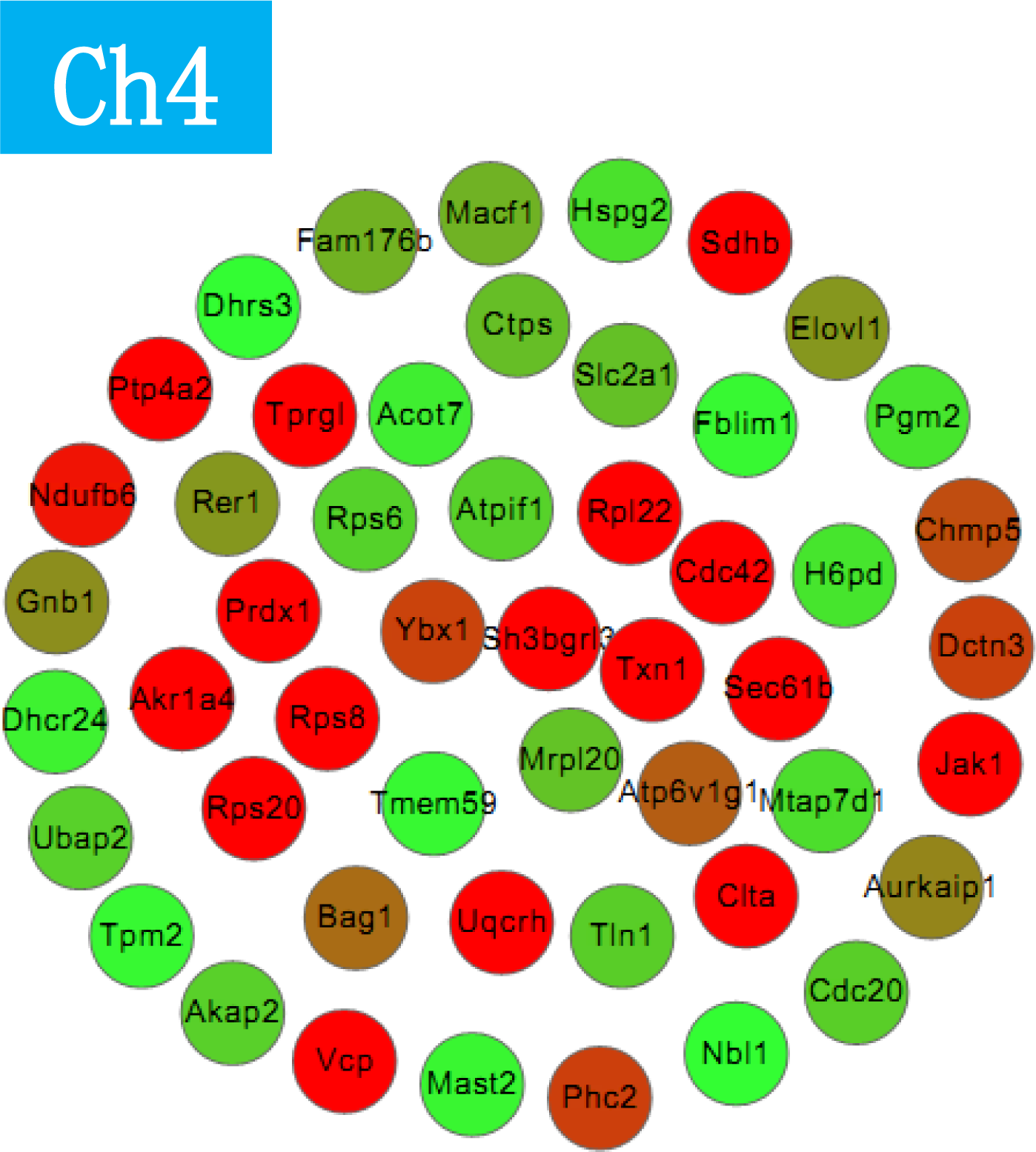

Supplement: Supplementary file 8 — Figure S8 Details of the selected core network genes in mouse CD8+ T cells come from bronchial lymph nodes in chromosome 4. [file JCMM-20-071-s008.tif]

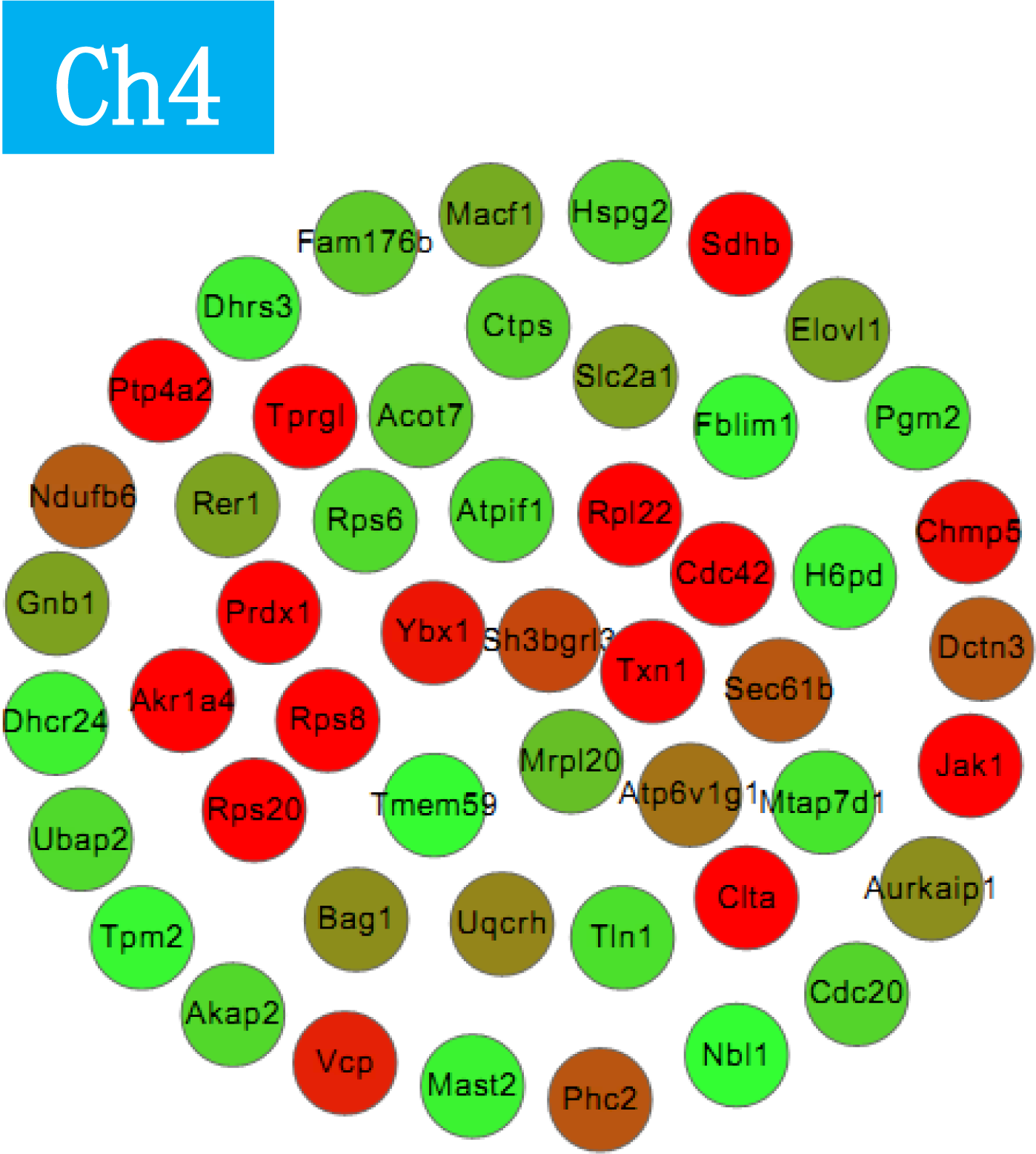

Supplement: Supplementary file 9 — Figure S9 Details of the selected core network genes in mouse CD8+ T cells from lung in chromosome 4. [file JCMM-20-071-s009.tif]
